# Poly(*N*-4-vinylbenzyl-1,4,7-triazacyclononane) Copper Complex Grafted Solid Catalyst for Oxidative Polymerization of 2,6-Dimethylphenol

**DOI:** 10.3390/molecules21020146

**Published:** 2016-01-26

**Authors:** Kei Saito, Koji Miyamoto, Sepa Nanayakkara, Hirotaka Ihara, Milton T. W. Hearn

**Affiliations:** 1 School of Chemistry, Monash University, Clayton, Victoria 3800, Australia; sepa.nanayakkara@monash.edu (S.N.); milton.hearn@monash.edu (M.T.W.H.); 2 Department of Applied Chemistry and Biochemistry, Kumamoto University, Kurokami, Kumamoto 860-8555, Japan; koji8342@gmail.com (K.M.); ihara@kumamoto-u.ac.jp (H.I.)

**Keywords:** solid catalysts, oxidative polymerization, macromolecular metal complexes

## Abstract

A new solid phase catalyst, poly(*N*-4-vinylbenzyl-1,4,7-triazacyclononane) copper(I) complex, grafted onto polystyrene particles, has been employed for the oxidative polymerization of 2,6-dimethylphenol using an aqueous biphasic (water/toluene) solvent system. The solid catalyst was synthesized by first grafting *N*-(4-vinylbenzyl)-1,4,7-triaza-cyclononane onto polystyrene particles using a radical mediated polymerization method and next by creating the polymer-metal complex of copper-triazacyclononane with these modified particles. Poly(2,6-dimethyl-1,4-phenylene oxide) was successfully obtained from the polymerization of 2,6-dimethylphenol using this new metal-organic solid phase catalyst.

## 1. Introduction

The fields of homogeneous and heterogeneous catalysis are often referred to as the “foundational pillars” of green chemistry [1]. In particular, the use of heterogeneous catalysts that can be recycled after their use is often favored for many chemical reactions within an industrial setting from the viewpoint of green chemistry [2]. One way to synthesize heterogeneous catalysts is to graft functional groups or polymers onto solid supports [3,4]. In this paper, we report the synthesis of poly(*N*-4-vinylbenzyl-1,4,7-triaza-cyclononane) (polyTACN) grafted polystyrene particles and their use as a polymeric metal complex solid catalyst to form poly(2,6-dimethyl-phenylene oxide) (PPO) from the oxidative polymerization of 2,6-dimethylphenol (DMP).

Since its discovery in 1959, PPO has become one of the most widely used engineering thermoplastics [5]. PPO is commonly synthesized by the oxidative polymerization of DMP. In current industrial processes, the oxidative polymerization of DMP is carried out at mild temperatures by passing oxygen through a solution of phenol in a non-polar solvent containing a copper-amine catalyst. Several copper-amine catalysts have been studied for this polymerization [6,7] with the more electron-donating amine ligands having higher catalytic activity for the oxidative polymerization of phenols [7]. In nature, tyrosinase is one of the enzymes that can oxidatively polymerize phenols. As tyrosinase models, the copper complexes of triazacyclononane (TACN) derivatives have been investigated as catalysts for the oxidative polymerization of phenols [8,9,10]. Kobayashi *et al.* for example, discovered that a copper TACN complex can be used as a catalyst for the highly regioselective oxidative polymerization of 4-phenoxyphenol [8]. Our group has previously reported the use of copper complexes of TACN derivatives as a catalyst in aqueous solutions for the oxidative polymerization of 2,6-difluorophenol and also as a catalyst for the oxidative polymerization of DMP using water as a solvent [9,10].

Solid phase catalysts have also been applied to the oxidative polymerization of phenols by several other groups of researchers. For example, Shentu *et al.* used polymer metal complexes, such as the Cu(II)-poly(*N*-vinylimidazole) complex, as a catalyst to form PPO in water [11]. These investigators also used Cu(II)-amine terminated poly(amidoamine) dendrimer complexes as a catalyst for the same polymerization [12]. Ueda *et al.* used a copper amine catalyst immobilized within mesoporous silica for the regioselective oxidative polymerization of phenols [13]. Hou *et al.* used Cu(II)-containing metal-organic frameworks (MOFs), based on heterocyclic ligand as a catalyst for the oxidative polymerization of DMP [14]. However, to date polymeric metal complexes of copper TACN derivatives have not been reported as a catalyst for the polymerization of DMP to form PPO.

Recently, we developed several polymer-grafted solid materials, such as poly(vinylsulfonic acid)-grafted polystyrene particles and 2,2,6,6-tetra-methylpiperidinyloxyl (TMEPO) radical polymer-grafted silicas, as acid and oxidation solid catalyst, respectively [15,16]. In this study, we synthesized composite polymer-grafted solid materials (PSt-polyTACN) by immobilizing polyTACN onto the surface of polystyrene particles. The synthetic intermediates were characterized by NMR, IR and elemental analysis. The copper complex of this polymeric solid, PSt-Cu(II)-polyTACN, was used as a catalyst for the oxidative polymerization of DMP to form PPO. An aqueous biphasic (water/toluene) solvent system was employed for this polymerization taking into consideration solvent selection according to the relevant green chemistry principles [17,18].

## 2. Results and Discussion

### 2.1. Synthesis of Poly(N-4-vinylbenzyl-1,4,7-triazacyclononane) Copper Complex Grafted Polystyrene Particle

**Scheme 1:**
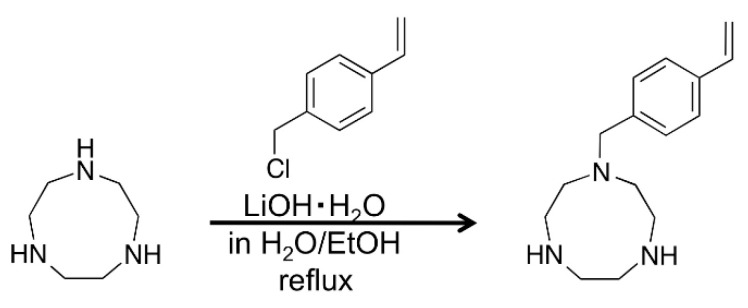
Synthetic scheme of vinylTACN synthesis.

TACN was synthesized using a previously reported method, based on modifications of the Rickman-Atkins reaction as adapted by Wieghardt *et al.* [19,20,21,22]. In brief, TACN was made by a condensation reaction from *N,N′,N*′′-tris(*p*-tolylsulfonyl)diethylenetriamine and 1,2-bis(*p*-tolyl-sulfonyloxy)ethane. The *N*-(4-vinylbenzyl)-1,4,7-triazacyclononane (vinylTACN) monomer for conversion to polyTACN, was synthesized by reacting vinylbenzyl chloride with TACN in the presence of LiOH·H_2_O in ethanol (Table 1). This reaction yielded mainly the required mono-substituted vinylTACN, with small amounts of the di-substituted and tri-substituted vinylTACN byproducts and trace amounts of a polymeric material that were removed by the column chromatography (Scheme 1).

**Table 1:** *N*-(4-Vinylbenzyl)-1,4,7-triazacyclononane (VinylTACN) synthesis (TACN/vinyl benzyl chloride = 5:1).

Entry	Reaction Time (h)	Temperature (°C)	Solvent	Yield (%)
1	5	reflux	ethanol/water	13
2	1	reflux	ethanol/water	27
3	3	r.t.	ethanol/water	25
4	1	reflux	THF	20

**Scheme 2:**
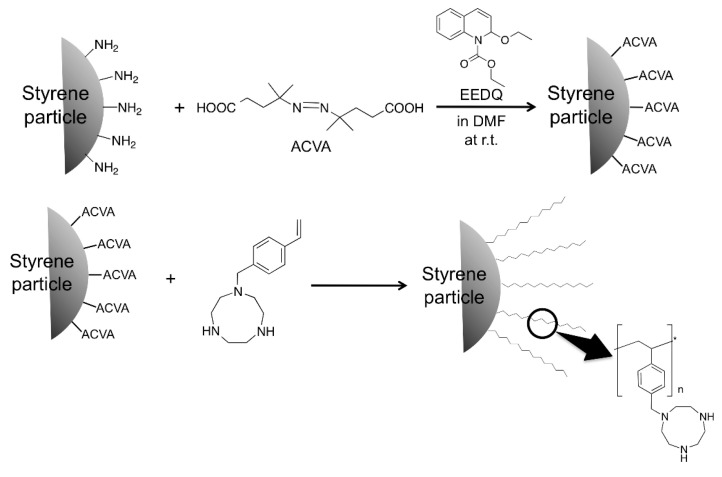
PolyTACN grafted PSt (PSt-polyTACN) synthesis.

To immobilize the vinylTACN polymer onto the surface of polystyrene (PSt) particles, first, a free radical initiator, 4,4′-azobis(4-cyanovaleic acid) (ACVA), was immobilized onto the surface of aminomethylated PSt beads using a condensation reaction with 2-ethoxy-1-ethoxycarbonyl-1,2-dihydroquinoline in DMF. Next, the ACVA-attached PSt was mixed with vinylTACN in the ratio (0.05:1 wt percentage) and heated at 90 °C for 8 h to allow polymerization to proceed, initiated by the radical initiator anchored onto the surface of PSt particles. Un-immobilized monomer and free polymer were then removed from the particles by washing with water, followed by methanol and finally acetone, to obtained polyTACN grafted PSt (PSt-polyTACN) as a solid light brown powder (Scheme 2).

### 2.2. Oxidative Polymerization of DMP to Form PPO Using PSt-polyTACN Copper Complex

**Scheme 3:**
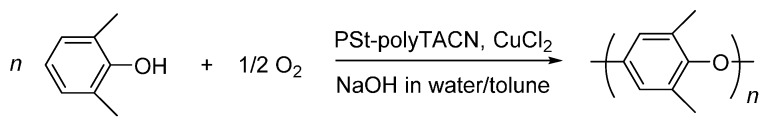
Oxidative polymerization of DMP using PSt-polyTACN copper complex.

**Figure 1:**
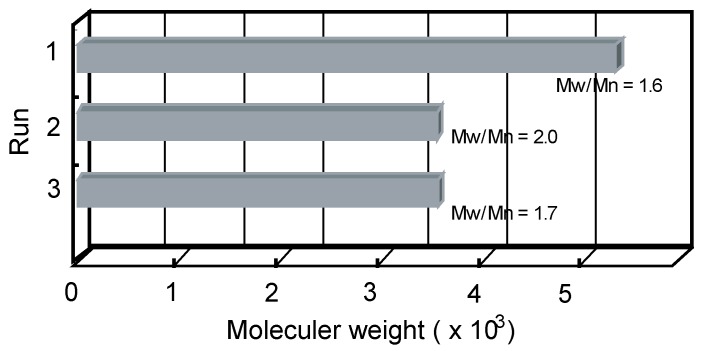
The molecular weight of PPO achieved from each run with recycling the recovered PSt-polyTACN copper complex as a catalyst in the oxidative polymerization of DMP.

In order to evaluate the functional effectiveness of the copper(II) complex of PS-polyTACN as an oxidative catalyst, DMP was selected as a suitable substrate since its oxidation occurs via an active phenoxy radical species, leading to polymerization. The copper complex of the obtained PSt-polyTACN material was used as the catalyst for the oxidative polymerization of DMP in an aqueous biphasic (water/toluene) solvent (Scheme 3). The water/toluene (1:1 *v*/*v*) system was chosen from green chemistry perspectives (e.g., the use of water as a benign solvent; the easy separation of the solid catalyst after the polymerization reaction was completed; and toluene as an efficient solvent for use in the DMP polymerization on the basis of its highly ranked attributes according to relevant green chemical solvent selection guidelines [17,18]. Accordingly, the UV-vis spectrum of PSt-polyTACN with CuCl_2_ present in toluene under an oxygen atmosphere was measured to confirm the formation of copper(II) complex in the solution under the same conditions that can be employed for the optimised large scale polymerisation reaction (Electronic Supporting Information Figure S1 for the spectrum). The polymerization procedure involved PSt-polyTACN, CuCl_2_, and a water/toluene solution of DMP, stirred under an oxygen gas envelop at 50 °C. Following recovery, the product so obtained after the polymerization was characterized by ^1^H-NMR and IR spectroscopy (^1^H-NMR (CDCl_3_), δ 2.09 (s, 6H, CH_3_), 6.47 (s, 2H, Ar-CH CH), IR (KBr): ν_C–O–C_ = 1186 cm^−1^) to confirm the PPO structure. The molecular weight of the obtained polymer was analyzed by gel permeation chromatography (GPC). The yield and molecular weight of the obtained PPO were 16% and *M*_w_ = 5100, *M*_w_/*M*_n_ = 1.6, respectively. The molecular weight of the polymer was comparable to the other PPO obtained in water or biphasic solvent system, however, the yield of the polymerization was lower than the other route due to the nature of the heterogeneous conditions with the solid catalyst [12,23]. The obtained PPO was evaluated by thermogravimetric analysis at a heating rate of 10 °C/min under a nitrogen atmosphere to measure the 10% thermal degradation temperature. The 10% thermal degradation temperature of PPO was determined to be 310 °C by thermogravimetric analysis [24,25]. In order to establish the recyclability of the catalyst, the solid catalyst was recovered by centrifugation and re-utilised in subsequent cycles. The catalyst was found to retain its activity and the yield of the polymerization product remaining constant even after the third cycle but the molecular weight of the obtained PPO was slightly reduced after the second cycle (Figure 1).

## 3. Experimental Section

### 3.1. Materials

All reagents and materials were purchased from Sigma-Aldrich (Sydney, Australia) and used without further purification.

### 3.2. Characterization

^1^H- and ^13^C-NMR spectra were recorded on a Bruker DPX-400 spectrometer. Infrared (IR) spectra were recorded on a Perkin-Elmer Spectrum RX1 FTIR spectrophotometer as KBr pellets. Ultraviolet-visible (UV-vis) spectra were recorded on a Cary 100Bio spectrophotometer as toluene solutions. Molecular weights of PPO were determined by GPC with a Tosoh high performance GPC system equipped with UV-8320 TSK GEL using Tosoh α 4000 and 2500 columns and DMF was used as the solvent. Polystyrene standards, TSK standard polystyrene purchased from TOSOH, were used to obtain calibration curves for the GPC. Thermal analysis of the polymer was performed using a Mettler Toledo TGA/DSC thermogravimetric and StarE software over a temperature range of 40–600 °C (heating rate: 10 °C/min^−1^ ) in N_2_ (flow rate: 20 mL/min^−1^).

### 3.3. Synthesis of 1,4,7-Triazacyclononane (TACN)

TACN was prepared following methods outlined in the literature [19,20,21,22].

### 3.4. Synthesis of N-(4-Vinylbenzyl)-1,4,7-triazacyclononane (VinylTACN)

A three necked flask was charged with TACN (5.0 g, 39 mmol), followed by the addition of deoxygenated ethanol (150 mL), LiOH·H_2_O (0.4 g, 9.5 mmol) in deoxygenated H_2_O (50 mL). The resulting solution was slightly cloudy and was allowed to stir at 50 °C for 30 min. 4-Vinylbenzyl chloride (1.1 g, 7.5 mmol) in ethanol was added dropwise to the mixture. The reaction mixture was refluxed under N_2_. After 2 h, the reaction mixture was concentrated to 50 mL on a rotary evaporator. Water was added to the residue and the solution was extracted 3-times with dichloromethane (30 mL). The combined dichloromethane extracts were washed with brine, dried with anhydrous Na_2_SO_4_, and concentrated under reduced pressure. The residue was loaded onto a silica column and eluted using chloroform–methanol (50:50, *v*/*v*, and 1% of triethylamine). Upon removal of the solvents, the chromatographically purified *N*-(4-vinylbenzyl)-1,4,7-triaza-cyclononane (0.5 g) was obtained as a light brown viscous oil and used immediately for the preparation of the grafted polystyrene particles. Typical recovered yields of the purified *N*-(4-vinylbenzyl)-1,4,7-triaza-cyclononane were 5%, ^1^H-NMR (CDCl_3_), δ (ppm), 2.65–3.1 (m, 12Η, ring CΗ_2_), 3.68 (s, 2H, benzyl CH_2_), 5.18 (d, 1Η, CΗ=CΗ_2_), 5.65 (d, 1Η, CΗ=CΗ_2_), 6.65 (dd, 1Η, CΗ=CΗ_2_), 7.20 (d, 2Η, aromatic CΗ), 7.30 (d, 2Η, aromatic CΗ), FT-IR (KBr) 3361, 2918, 2849, 1654, 1559 cm^−1^.

### 3.5. Synthesis of Poly(N-4-vinylbenzyl-1,4,7-triazacyclononane) Grafted Polystyrene Particles (PSt-polyTACN)

4,4-Azobis(4-cyanovaleric acid) (ACV, 4.50 g, 16 mmol) and (1-ethoxycarbonyl)-2-ethoxy-1,2-di-hydroquinoline (EEDQ, 7.91 g, 32 mmol) were dissolved in DMF (200 mL). N_2_ gas was bubbled through the solution at room temperature for 30 min. Aminomethylated polystyrene beads (4.0 mmol g-1 NH2 loading, 2 g) were then added to the solution, and N_2_ gas was bubbled through the mixture at room temperature again for 30 min before the reaction. The reaction was carried out at 25 °C for 8 h under N_2_ atmosphere. The ACV-attached PSt beads were washed with DMF and ethanol, consecutively, and dried *in vacuo*. The ACV-attached PSt beads (3 g) were obtained as a faint yellow powder. Next, the *N*-(4-vinylbenzyl)-1,4,7-triaza-cyclononane (0.5 g, 2.04 mmol) and the ACV-attached PSt beads (25 mg) suspended in methanol (50 mL) were added to a round bottom flask. The reaction was carried out at 90 °C for 8 h under N_2_ atmosphere. The PSt-TACN beads were filtered and extensively washed three times with water then methanol, and finally with acetone by decantation so as to remove any un-immobilized polymer and monomers. The product was dried *in vacuo* for 1 day. The PSt-TACN beads (29 mg) were obtained as a brownish powder. FT-IR (KBr) 1653, 1446, 1349, 1011 cm^−1^, Elemental analysis: % C 72.5, % H 7.51, % N 6.48.

### 3.6. Oxidative Polymerization of 2,6-Dimethyl Phenol (DMP) in Water by Solid State Catalyst

DMP (1.22 g, 10 mmol) and NaOH (0.4 g, 10 mmol) were dissolved in water (25 mL) and the aqueous solution added to a toluene solution (25 mL) of PolyTACN (45 mg) and CuCl_2_ (2.2 mg, 0.0162 mmol). The mixture was then vigorously stirred under an oxygen atmosphere at 50 °C for 6 h. The organic layer was separated and concentrated to 10 mL *in vacuo*, and then added dropwise to methanol (200 mL) containing a few drops of conc. HCl. The product was obtained by filtration. Yield: 16%. ^1^H-NMR (CDCl_3_), δ 2.09 (s, 6H, CH_3_), 6.47 (s, 2H, Ar-CH CH), FT-IR (KBr) 3361, 2918, 2849, 1654, 1559, 1184 cm^−1^*. M*_w_ = 5100, *M*_w_/*M*_n_ = 1.6.

## 4. Conclusions

This study has documented the synthesis of PSt-polyTACN by graft polymerization of vinylTACN onto the surface of PS. The obtained PSt-polyTACN was used as a solid ligand material to prepare the PSt-polyTACN copper complex catalyst, which in turn was employed for the oxidative polymerisation of DMP in a water/toluene biphasic system to form PPO. The obtained polymer was characterized by ^1^H-NMR, IR spectroscopy, GPC and TGA to confirm the PPO structure and thermal stability. The obtained PPO was found to be comparable to the PPO obtained using other water or biphasic solvent systems, however, the yield of the polymerization was lower than the other routes due to the nature of the heterogeneous conditions using the solid catalyst. The oxidative polymerization achieved with this new solid state catalyst thus documents its potential to be employed in polymerization reactions based on biphasic water-organic solvent combinations with the organic solvent chosen according to green chemical metrics.

## Supplementary Materials

Supplementary materials can be accessed at: http://www.mdpi.com/1420-3049/21/2/146/s1.
