# Supplementary material for: Poly(N-4-vinylbenzyl-1,4,7-triazacyclononane) Copper Complex Grafted Solid Catalyst for Oxidative Polymerization of 2,6-Dimethylphenol
Source: Molecules. 2016 Jan 26;21(2):146. doi: 10.3390/molecules21020146 (PMC6273911; doi:10.3390/molecules21020146)
Supplement: Supplementary file 1 [file molecules-21-00146-s001.pdf]

Supplementary Materials: Poly(*N*-4-vinylbenzyl-1,4,7-triazacyclononane) Copper Complex Grafted Solid Catalyst for Oxidative Polymerization of 2,6-Dimethylphenol

Kei Saito, Koji Miyamoto, Sepa Nanayakkara, Hirotaka Ihara and Milton T. W. Hearn

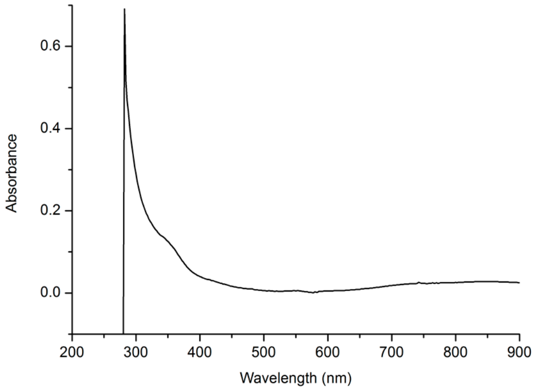

Figure S1. UV-vis spectrum of the PSt-polyTACN with $CuCl_2$ present in toluene.

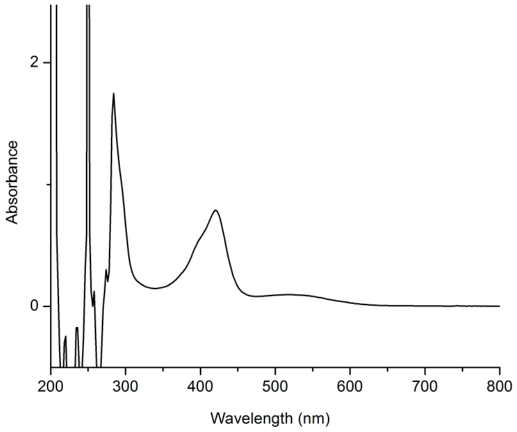

Figure S2. UV-vis spectrum of the polymerization solution.

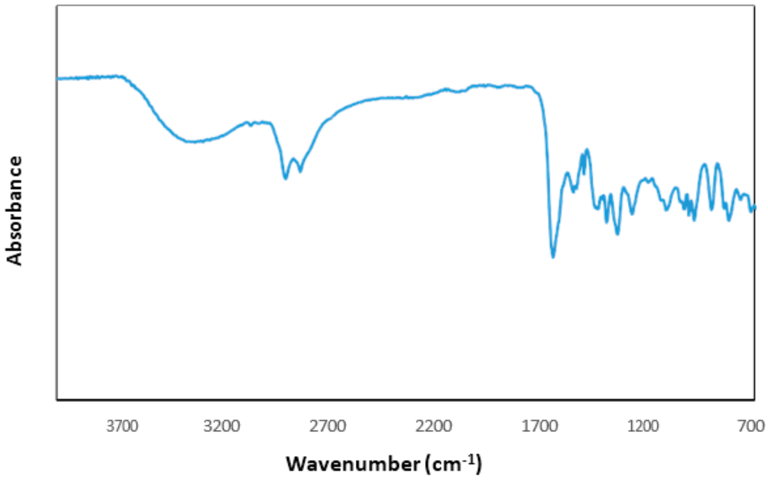

Figure S3. FT-IR spectrum of the obtained PPO.

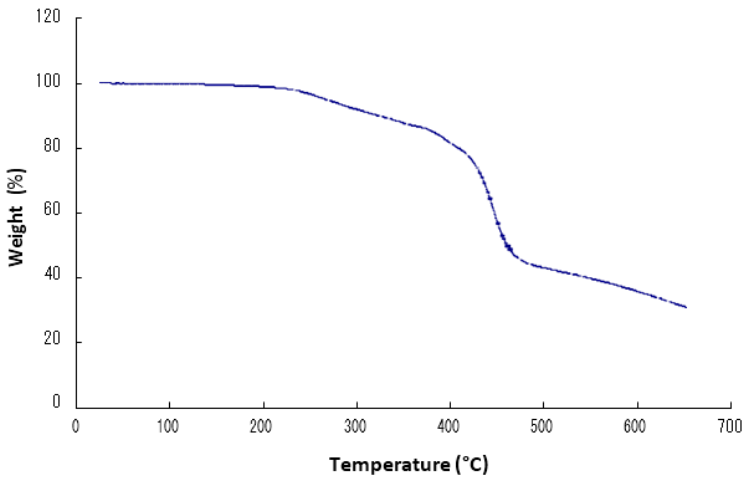

Figure S4. TGA result for the obtained PPO.
